# Treatment modalities for granulomatous mastitis, seeking the most appropriate treatment with the least recurrence rate: a systematic review and meta-analysis

**DOI:** 10.1186/s40001-024-01761-3

**Published:** 2024-03-12

**Authors:** Roham Sarmadian, Fatemeh Safi, Hossein Sarmadian, Maryam Shokrpour, Amir Almasi-Hashiani

**Affiliations:** 1 Student Research Committee, Tehran University of Medical Sciences, Tehran, Iran; 2 Department of Radiology, School of Medicine, Arak University of Medical Sciences, Arak, Iran; 3 Department of Infectious Diseases, School of Medicine, Arak University of Medical Sciences, Arak, Iran; 4 Department of Gynecology, School of Medicine, Arak University of Medical Sciences, Arak, Iran; 5 Department of Epidemiology, School of Health, Arak University of Medical Sciences, Basij Square, Arak, Iran; 6 Traditional and Complementary Medicine Research Center (TCMRC), Arak University of Medical Sciences, Arak, Iran

**Keywords:** Granulomatous mastitis, Recurrence, Therapeutic modalities, Surgery, Conservative treatment, Observation, Combination therapy, Granulomatous lobular mastitis

## Abstract

**Background:**

Granulomatous mastitis (GM) is a rare, benign, inflammatory breast disease with an unknown etiology that predominantly affects women of reproductive age. The definitive treatment of GM is currently controversial; an appropriate therapeutic strategy has yet to be identified, and the disease’s high recurrence rate remains. This study aims to determine the recurrence rate for each GM treatment strategy to identify the most appropriate treatment modality.

**Methods:**

The search for relevant articles was undertaken using three international databases, including Medline, Scopus, and Web of Science. Articles published in English until the end of 2021 evaluating the recurrence rate of GM were included. Using Stata 13.0, the pooled incidence and 95% confidence interval (CI) for the recurrence rate were determined.

**Results:**

Sixty-five eligible studies were included in our study. The recurrence rates of systemic steroid use, topical steroid use, antibiotic use, methotrexate use, observation, drainage, excision, antibiotic use and surgery, steroid use and surgery, antibiotic and steroid use, methotrexate and steroid use were 24% (95% CI: 21–27%), 11% (95% CI: 6–21%), 18% (95% CI: 14–22%), 13% (95% CI: 7–22%), 11% (95% CI: 7–17%), 65% (95% CI: 50–78%), 13% (95% CI: 10–16%), 23% (95% CI: 14–36%), 7% (95% CI: 5–11%), 11% (95% CI: 6–18%), and 4% (95% CI: 2–8%), respectively. Drainage had the highest recurrence rate, while combined methotrexate and steroid treatment had the lowest rate.

**Conclusion:**

The optimal treatment strategy for GM depends on the disease’s severity, consequences, and the patient's features. The study results indicate that combination therapy is preferable for minimizing the risk of relapse and reducing treatment complications.

## Backgrounds

Granulomatous mastitis (GM) is an uncommon, benign, inflammatory breast disease with an unknown etiology that mainly affects women of childbearing age [1]. Clinical manifestations, including swelling, mass, fistula formation, and radiological findings such as abscess, lymph node enlargement, calcifications, focal or diffuse asymmetric density, and hypoechoic lesions, can lead to misdiagnosis as carcinoma or infection [2, 3]. There are no distinguishing imaging findings for GM and breast cancer in any imaging modality [3]. Histological evaluation is usually used to arrive at a definitive diagnosis of GM. Giant cells, epithelioid histiocytes, non-caseating granulomas, lobulocentric granulomatous inflammation, and neutrophils are all common characteristics of GM [4].

There are two types of GM: Idiopathic GM (IGM) and specific GM (SGM). SGM is a rare secondary complication of tuberculosis, sarcoidosis, Wegener's granulomatosis, syphilis, corynebacterial infection, foreign body reaction, etc. IGM is defined as GM without any other identifiable causes [5]. However, autoimmune response, infection, and hormonal disruption are the three leading hypotheses for the etiology of IGM, with the autoimmune response hypothesis being the most widely accepted [6]. In areas with a high prevalence of tuberculosis, tuberculous mastitis must be ruled out before confirming IGM [7, 8].

The definitive treatment of GM is controversial; an optimal treatment strategy has not yet been determined, and the high recurrence rate persists [9]. If the underlying cause of the granulomatous inflammation is diagnosed, the treatment is based on the underlying cause [10]. Otherwise, conventional GM treatment involves close follow-up, invasive methods like drainage, and wide excision, as well as a variety of conservative methods such as extensive courses of antibiotics, systemic or topical steroids, and immunosuppressive therapy such as methotrexate (MTX) [11, 12]. Due to the unfavorable results associated with surgical therapies for GM, including poor wound healing, fistula formation, abscess formation, and Recurrence, corticosteroids appeared helpful in reducing adverse outcomes such as mastectomy [13]. Treatment with oral corticosteroids usually takes at least 2 to 3 months and might cause significant adverse effects, with the chance of Recurrence remaining [14]. The recurrence rate of GM may reach 50% following restricted surgical excision and 16–50% following systemic corticosteroids [15]. Researchers are now looking into combining treatments to prevent the relapses that can occur with monotherapy [16]. However, the efficacy of this method has not yet been thoroughly evaluated.

Only few studies have been undertaken to compare the various treatments for this disease. This study aims to determine the recurrence rate for each GM treatment approach. In this manner, the disease can be treated using the most effective treatment with the lowest recurrence rate.

## Methods

### Study design

This research consisted of a systematic review and meta-analysis. This was accomplished using the Preferred Reporting Items for Systematic reviews and Meta-Analyses (PRISMA) guideline and the Cochrane Handbook for Systematic Reviews of Interventions. This study was approved by the Ethical Committee of Arak University of Medical Sciences (Code: IR.ARAKMU.REC.1401.114). This study did not include the human sample, and previously published studies were examined. Therefore, informed consent was not used in this study.

### Search strategy

English-language articles published up until the end of 2021 were searched. The search for relevant publications was conducted using various keywords for three international databases, including Medline via PubMed, Scopus, and Web of Science. To retrieve publications, the following search query was performed, and the search was narrowed to only human and English full-text studies:

(““Granulomatous Mastitis”“[Title/Abstract] OR ““Granulomatous Mastitis”“[Text Word] OR ““Granulomatous Mastitis”“[MeSH Terms]) AND (““Recurrence”“[MeSH Terms] OR ““Recurrence”“[Text Word] OR ““Recurrence”“[Title/Abstract] OR ““recurr*”“[Text Word] OR ““relapse”“[Text Word])) AND (english[Filter])”.

### Study selection

To select appropriate articles, retrieved articles were entered into Endnote software, and duplicate articles were removed. The titles and abstracts of the remaining articles were then screened, and irrelevant articles were discarded. Moreover, any review articles without introducing new cases, letters to editor, case reports and conference abstracts were excluded from the study. Afterward, the full text of the remaining articles was reviewed, and articles lacking desired information were excluded. Finally, the required information was extracted from the relevant articles.

### Inclusion and exclusion criteria

English-language original articles published up through the end of 2021 that evaluated the recurrence rate among GM patients met the eligibility criteria. There was no time limit on the entry of the articles. Any reported recurrence, whether radiological or biopsy-proven, was considered valid for this study.

GM is classified as mild when there are a few localized granulomas and severe when there is extensive involvement, leading to significant inflammation, tissue damage, and more pronounced symptoms, including severe breast pain, swelling, skin changes, nipple retraction, and the formation of draining sinuses or abscesses [17, 18]. In light of this, studies have reported on various treatments, including topical and oral medications, injections, and surgical interventions. In our research, all disease severity levels and the majority of utilized medications have been included. Articles that only had information about incomplete disease resolution or reported recurrence of a treatment method that was not relevant to at least one of our therapeutic options (systemic steroid use, topical steroid use, antibiotic use, MTX use, observation, drainage, excision, antibiotic use and surgery, steroid use and surgery, antibiotic and steroid use, MTX and steroid use) were excluded from our study. There was no minimum follow-up duration requirement for studies to be included to assess for recurrence. However, it should be noted that studies with an extremely short follow-up period may fail to detect recurrences.

In instances when the article was relevant but lacked the required information, the author was contacted. Two individuals separately completed each step of the selection process. In instances of disagreement, decisions were made in collaboration with the other authors.

### Data extraction

The extracted data from each article included the name of the first author, the year the article was published, the sample size, the type of disease, the type of treatment administered, the country of the study, the rate of Recurrence following each treatment method (surgery/pharmacotherapy), the follow-up duration, and the quality score of the studies.

### Risk of bias

The quality assessment of the retrieved studies was performed by two authors using the quality assessment checklist for prevalence studies, which is adapted from Hoy et al. [19] (Appendix 1). The score of this checklist ranged from 0 to 9, which divides the articles into three groups, including low risk (score 0–3), moderate risk (score 4–6), and high risk (score 7–9).

### Statistical analysis

To test for heterogeneity among the studies, the I^2^ statistic was calculated using the chi-square test; if there was substantial heterogeneity among the studies, the reason for the heterogeneity was investigated using meta-regression and subgroup analysis. In the case of heterogeneity, a random effect model was utilized to combine data. Since there was no evidence in favor of heterogeneity among our data, the fixed-effect model was used to pool the data in all cases. Begg’s test was utilized to examine publication bias. In routine meta-analysis packages, when the disease prevalence is 0 or 100 because the software cannot calculate the standard deviation, it excludes the mentioned study from the analysis, leading to overestimation or underestimation of the desired index. Therefore, to pool the results of primary studies, we used the “metapreg” package in Stata [20], in which the Agresti-Coull method was used to estimate the confidence interval. All analyses were done using Stata software version 16 (Stata Corp, College Station, TX, USA).

Lastly, In addition to discussing the appropriate circumstances for the use of each aforementioned therapeutic modality, a discussion on rifampicin’s application in granulomatous lobular mastitis (GLM) which has recently attracted attention, has been attempted.

## Results

### Study selection and study characteristics

**Fig. 1:**
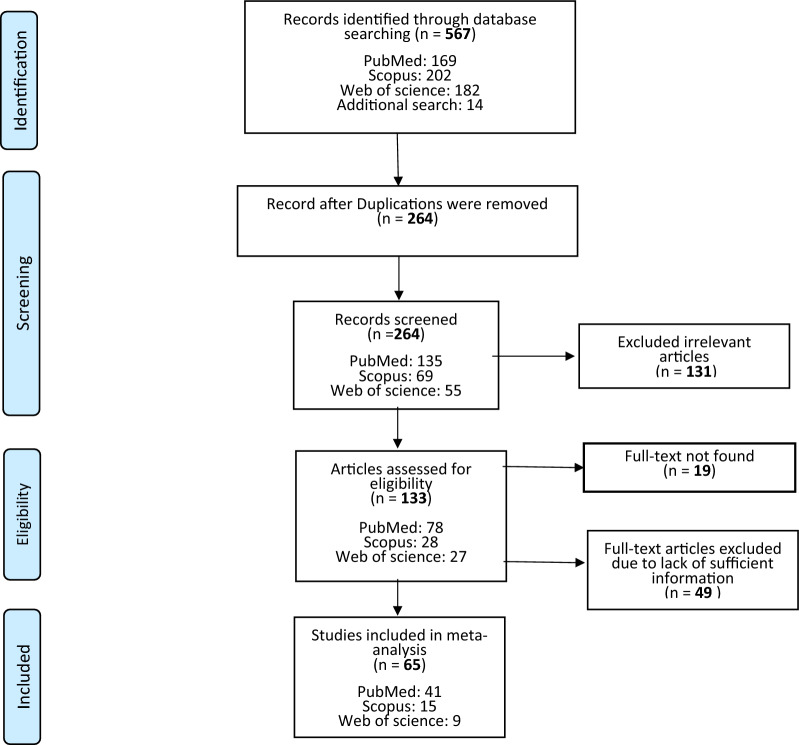
Flow diagram of the literature search for studies included in the meta-analysis

The process of article searching and screening is displayed in Fig. 1. We searched the three international databases to find the relative papers and retrieved 553 papers (Scopus: 202, PubMed/Medline: 169, Web of Science: 182). In addition to the mentioned databases, we searched Google Scholar to find the gray literature, and the references of the final selected papers were searched manually (*n* = 14).

As shown in Fig. 1, out of 567 retrieved papers, after deleting the 303 duplicate papers, the title and abstract of 264 papers were screened, and 131 irrelevant papers were excluded. In the next step, the full texts of the remaining 133 studies were screened in detail. Finally, 68 studies were excluded, 65 met the inclusion criteria, and their required data were extracted and included in the meta-analysis.

The details of the included studies are presented in Table 1. The earliest included study was published in 1989, and the most recent in 2021. Turkey had the most articles among countries in the world. The sample size ranged from 10 to 716 participants. The non-random sampling method has been the most common in selecting samples, and as shown in Table 1, most studies were conducted on Idiopathic granulomatous mastitis (IGM).

**Table 1:** Characteristics of the included studies

ID	Author	Year	Country	Sample size	Sampling	Age(years)	Treatments (Recurrence)	Follow_up	Type	Risk of bias	References
1	Akbulut S	2011	Turkey	4	Non-Randomized	30–42	Oral steroid + MTX (0/4)	2–9 months	IGM	8	[21]
2	Akcan A	2014	Turkey	74	Non-Randomized	36.6	Excision (4/51), Steroid + surgery (0/21)	3–170 months	IGM	3	[22]
3	Al-Jarrah A	2013	Oman	20	Non-Randomized	37.5	Antibiotic (0/19), Excision (0/1)	11–33 months	IGM	5	[23]
4	Alrayes A	2019	Bahrain	18	Non-Randomized	39	Excision (0/18)	12‐24 months	IGM	6	[1]
5	Altintoprak F	2015	Turkey	28	Non-Randomized	35.7	Topical steroid (3/28)	12–72 months	IGM	5	[24]
6	Aslan H	2018	Turkey	39	Non-Randomized	Conservative:38.44 ± 9.6 surgery:36.05 ± 7.44	Antibiotic (4/18), Mastectomy (1/21)	conservative: 9.05 ± 14.5 months surgery:10.28 ± 10.08 months	IGM	3	[3]
7	Asoglu O	2005	Turkey	18	Non-Randomized	41.5 (16–80)	Antibiotic + surgery (1/18)	36 (6–60 months)	IGM	6	[25]
8	Atalay C	2011	Turkey	51	Non-Randomized	33 (22–57)	Excision (3/50), Mastectomy (0/1)	38 (12–58 months)	IGM	3	[26]
9	Azlina, A. F	2003	Malaysia	12	Non-Randomized	36.5	Oral steroid (6/12)	2–6 months	CGM	8	[27]
10	Baslaim M. M	2007	Saudi Arabia	17	Non-Randomized	34 (21–45 years)	Antibiotic + surgery (0/17)	24 (15–42 months)	IGM	6	[28]
11	Bouton M. E	2015	USA	27	Non-Randomized	33	Observation (3/27), Excision (1/9)	7.4 (6–24 months)	IGM	6	[29]
12	Calis H	2014	Turkey	13	Non-Randomized	44 (25–77)	Oral steroid (2/12), Mastectomy (0/1)	24 months	IGM	6	[17]
13	Cetinkaya G	2021	Turkey	118	Non-Randomized	35 (21–65)	Oral steroid (8/36), Antibiotic (4/21), Observation (1/50), Imuran (0/2)	70 (16–124 months)	IGM	4	[30]
14	Chirappapha P	2018	Thailand	36	Non-Randomized	38 (21–81)	Oral steroid (1/6), Drainag (3/7), Excision (8/23)	20.73 (1.26–118.8 months)	IGM	3	[31]
15	Co M	2018	China	88	Non-Randomized	33 (20–54)	Oral steroid (9/62), Antibiotic (4/22), Excision (1/4)	14 (4–51 months)	IGM	6	[32]
16	Cornejo-Juarez, P	2014	Mexico	58	Non-Randomized	38 ± 12	Oral steroid (2/8), Antibiotic (6/20), Antibiotic + steroid (3/20), Observation (3/7), Excision (1/3)	16.7 ± 13.8 months	IGM	3	[33]
17	Dag A	2021	Turkey	18	Non-Randomized	34.2(25–42)	Excision + oncoplastic breast surgery (2/18)	20 (6–44 months)	IGM	6	[34]
18	Elzahaby I. A	2016	Egypt	30	Non-Randomized	33(23–43)	Excision (0/30)	19 (8–44 months)	IGM	7	[35]
19	Erozgen F	2010	Turkey	27	Non-Randomized	35.3 (22–52)	Oral steroid (0/4), Drainage + steroid (1/14), Excision + steroid (0/9)	7 (1–48 months)	IGM	6	[36]
20	Erturk T. F	2021	Turkey	86	Non-Randomized	Conservative:37.2 (23–63) surgery:36.8 (24–65)	Intralesional injection of steroid + topical steroid (0/38), Excision (15/48)	12-month	IGM	4	[37]
21	Gopalakrishnan Nair C	2014	India	22	Randomized	32.85	Oral steroid (1/22)	24 months	IGM	5	[38]
22	Govindasamy A	2016	India	26	Non-Randomized	20–53	Excision (0/26)	12–84 months	GM	6	[39]
23	Hur S. M	2013	Korea	50	Non-Randomized	37.1 ± 7.9	Oral steroid (9/13), Antibiotic (2/3), Observation (1/8), Drainage (10/14), Excision (1/12)	32.0 ± 18.1 months	GLM	4	[40]
24	Kafadar M. T	2021	Turkey	17	Non-Randomized	40.4 ± 5.3	Oral steroid + MTX (4/17)	2–3 months of treatment	IGM	8	[41]
25	Karanlik H	2014	Turkey	60	Randomized	35 (18–58)	Oral steroid (7/23), Steroid + surgical (0/37)	conservative:12 (2–18) months surgery + conservative:38 (22–78 months)	IGM	2	[42]
26	Kaviani A	2018	Iran	374	Non-Randomized	34.06 ± 6.7	Oral steroid (39/142), antibiotic (22/154), MTX (1/13), NSAID (26/140), Observation (10/66), Surgical procedures (89/158)	–	IGM	6	[43]
27	Kayahan M	2012	Turkey	31	Non-Randomized	35 (27–62)	Oral steroid (1/12), Antituberculosis (0/1), Drainage (3/6), Excision (1/12)	27.8 (9–124 months)	GM	5	[44]
28	Kehribar D. Y	2020	Turkey	33	Non-Randomized	38.64 ± 6.9	Oral steroid + MTX (0/33)	24 months	IGM	6	[45]
29	Koksal H	2021	Turkey	108	Non-Randomized	35.5 (21–68)	Oral steroid (2/18), Wait-and-watch or only antibiotic (3/42), Surgical procedures (5/48)	Up to 20 months	IGM	4	[46]
30	Liao H	2020	China	28	Non-Randomized	32 (26–43)	Surgical procedures (1/24)	20 (11–40 months)	GLM	6	[47]
31	Mizrakli T	2014	Turkey	49	Non-Randomized	34.3 ± 4.37	Antibiotic (0/1), Oral steroid + MTX (0/40), Antituberculosis (0/5), NSAID (0/2), Excision (0/1)	At least 6 months	GM	6	[48]
32	Nair C. G	2015	India	23	Non-Randomized	33.42	Antibiotic + steroid (1/23)	24 months	IGM	5	[49]
33	Ocal K	2010	Turkey	16	Non-Randomized	34 (24–51)	Excision (3/16)	24 (6–36 months)	GM	5	[50]
34	Ozel L	2012	Turkey	8	Non-Randomized	37( 27–48)	Antibiotic + surgery (2/8)	12 months	GM	8	[51]
35	Papila Kundaktepe B	2021	Turkey	60	Non-Randomized	32.77 ± 6.03 (23–49)	MTX (8/60)	831 ± 547 days	IGM	5	[51]
36	Ringsted S	2021	Oregon	20	Non-Randomized	32(16–42)	Oral steroid (5/12), MTX (0/5), Steroid + surgery (0/3)	27 (5–63 months)	IGM	6	[52]
37	Salehi M	2014	Iran	59	Non-Randomized	conservative:30.3 ± 6.38 surgery:36.74 ± 13.51	Antibiotic + steroid (1/20), Partial mastectomy (32/39)	12 months	IGM	6	[53]
38	Sen Oran E	2013	Turkey	46	Non-Randomized	33 (28–55)	Oral steroid (5/25), Excision (3/18), Steroid + surgery (0/3)	35.4 (3–135 months)	IGM	3	[54]
39	Shin Y. D	2017	South Korea	34	Non-Randomized	37(24—57)	Excision (5/20), steroid + drainage (1/14)	45.5 (22–98 months)	GLM	4	[55]
40	Shojaee L	2021	Iran	87	Non-Randomized	34	Oral steroid (9/23), Excision (7/17), steroid + drainage (9/47)	26 (8–48 months)	IGM	3	[56]
41	Skandarajah, A	2015	Australia	11	Non-Randomized	40	Oral steroid (1/3), Antibiotic + surgery (3/5)	6 (3 months–15 years)	IGM	8	[57]
42	Tan Q. T	2019	Singapore	113	Non-Randomized	36.2(25–63)	Oral steroid (16/73), Antibiotic (19/79), MTX (1/1), Observation (0/5), Surgical procedures (6/24)	252 days	GM	5	[4]
43	Tekgoz E	2020	Turkey	53	Non-Randomized	37.2 ± 6.6	Oral steroid (0/3), Oral steroid + MTX (1/41), Azathioprine + steroid (0/3), Observation (0/1), Excision (3/3), MTX + surgery (0/2)	13.83 (1.61–100.83 months)	IGM	6	[2]
44	Toktas O	2021	Turkey	78	Non-Randomized	36.7 ± 1.4	Oral steroid (15/32), Intralesional injection of steroid + topical steroid (4/46)	23.2 ± 9.1	IGM	4	[6]
45	Velidedeoglu M	2016	Turkey	10	Non-Randomized	38.4 ± 8.3(29–52)	Antibiotic (0/1), Drainage + antibiotic (1/5), Drainage + antibiotic + steroid (0/4)	21 (11–26 months)	IGM	8	[58]
46	Wang J	2021	China	200	Non-Randomized	38	Oral steroid (24/104), Surgical procedures (8/156)	15.64 (12–36months)	IGM	3	[59]
47	Yabanoglu H	2015	Turkey	77	Non-Randomized	Surgery:36 (28–68) Conservative:37 (28–59)	Oral steroid (9/44), Excision (0/31), Mastectomy (0/2)	16.57 ± 18.57	IGM	4	[60]
48	Yau F. M	2010	Canada	11	Non-Randomized	37.4 (23–49)	Antibiotic + surgery (8/11)	18.6 months	GM	6	[61]
49	Zhang X	2020	China	53	Non-Randomized	34.6 ± 5.9	Excision (4/25), Surgery + traditional chinese medicine (Yanghe decoction) (0/28)	13.2 ± 10.0 months	IGM	5	[14]
50	Zhang X	2020	China	68	Non-Randomized	35 (22–55)	Excision (3/68)	24 months	IGM	6	[62]
51	Ahmed, Y. S	2016	Egypt	13	Non-Randomized	35.53 ± 7.25 (27–51)	Excision (2/13)	–	IGM	7	[63]
52	Akin M	2017	Turkey	11	Non-Randomized	35.5 (29–45)	Antibiotic + steroid (0/11)	60 (16–110 months)	IGM	7	[64]
53	Atak T	2015	Turkey	50	Non-Randomized	39.07 ± 11.5	Oral steroid (1/6), Antibiotic + anti inflammatory agent (1/11), Drainage (12/16), Excision (1/7)	24.85 ± 19.7 months	IGM	5	[65]
54	Basim P	2021	Turkey	122	Non-Randomized	34.1 (29–47)	Oral steroid (8/28), Excision (6/19), Steroid + surgery (5/75)	32.5 (19–67 months)	IGM	3	[16]
55	Cetin K	2019	Turkey	124	Randomized	33.9 ± 6.8 (20–58)	Oral steroid (6/42), Topical steroid (5/42), Topical + oral steroid (6/40)	94 ± 28 (55–191 weeks)	GM	3	[66]
56	Emre A	2018	Turkey	32	Non-Randomized	40.3 ± 10.7(26–70)	Oral steroid (1/3), Antibiotic (2/10), Antibiotic + steroid (1/9), Anti TB + antibiotic (0/2), Anti TB (0/1), Antibiotic + chemotherapy (0/1), Observation (1/6)	687 ± 618 (5–1800 days)	IGM	7	[67]
57	Galea M. H	1989	England	6	Non-Randomized	34	Excision (2/6)	–	GLM	8	[68]
58	McLean, N. R	2019	United Kingdom	4	Non-Randomized	47.5 (26–62)	Antibiotic + surgery (0/1), Steroid + antibiotic + mastectomy (0/3)	30 months	IGM	8	[69]
59	Oak J	2021	India	40	Non-Randomized	33	Oral steroid + MTX (2/40)	12 months	IGM	5	[70]
60	Oran E. S	2013	Turkey	46	Non-Randomized	33 (28–55)	Oral steroid (5/25), Excision (3/18), Steroid + surgery (0/3)	35.4 (3–135 months)	IGM	4	[71]
61	Pandey T. S	2021	USA	49	Non-Randomized	35 (24–67)	Oral steroid (10/44), Observation (0/3), Excision (0/2)	6–12 months	IGM	5	[72]
62	Postolova A	2020	USA	19	Non-Randomized	33.5	Antibiotic (3/19)	36 (12–84 months)	IGM	5	[73]
63	Sheybani F	2015	Iran	22	Non-Randomized	32.82 ± 6.26 (23–47)	Oral steroid (3/15), MTX (0/1), Oral steroid + MTX (0/6)	11.91 ± 4.4 (6–22)	IGM	5	[7]
64	Avci M. E	2015	Turkey	9	Non-Randomized	21–39	Oral steroid (8/9)	–	IGM	8	[74]
65	Seo H. R. N	2012	Korea	IGM:58 TM:10	Non-Randomized	IGM:33.5 TM:40	Antibiotic + steroid (5/21), Antituberculosis (1/10), Excision (0/48)	IGM:11.71 TM:21	IGM & TM	7	[75]

*GM* granulomatous mastitis, *IGM* idiopathic granulomatous mastitis, *CGM* chronic granulomatous mastitis, *GLM* granulomatous lobular mastitis, *TM* tuberculous mastitis, *MTX* methotrexate, *NSAID* nonsteroidal anti-inflammatory drug

### Risk of bias within studies

We used a quality assessment checklist for prevalence studies to assess the risk of bias within studies. The quality assessment results revealed that 16.92% of studies were low risk, 63.08% were moderate risk, and 20% were high risk.

### Quantitative data synthesis and heterogeneity across studies

#### The recurrence rate following oral steroid use

**Fig. 2:**
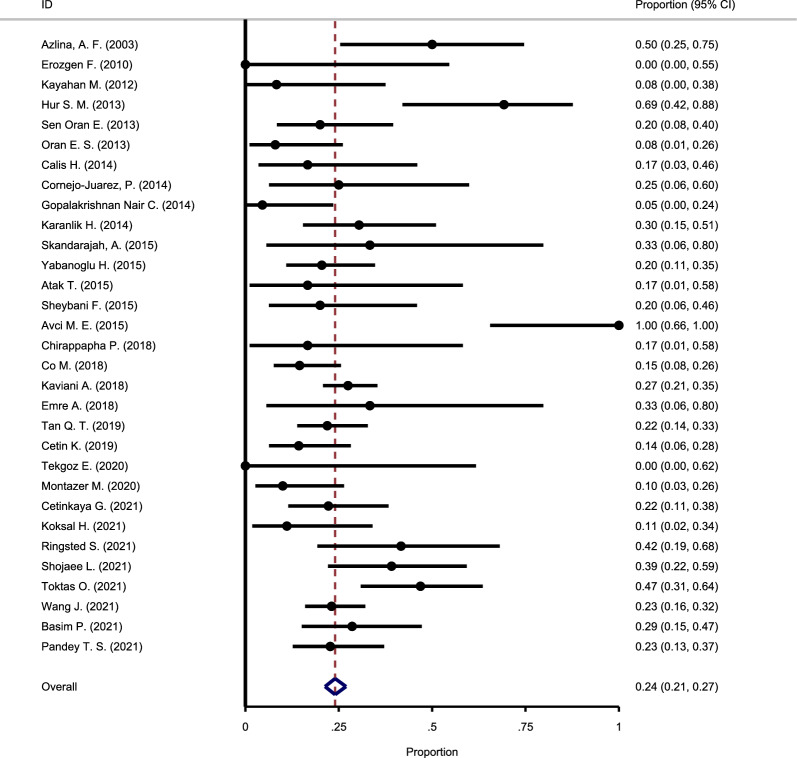
Forest plot showing the recurrence rate following oral steroid use

To estimate the recurrence rate following oral steroids, 31 primary studies were included in the meta-analysis. The chi-square test results suggested no substantial heterogeneity among studies (Chi square = 16.3, I-square = 46.99%), and the fixed-effect model was used to pool the reported results. The meta-analysis results estimated the pooled recurrence rate as 24% (95% CI: 21–27%) (Fig. 2).

#### The recurrence rate following topical steroid use

Only two studies investigated the recurrence rate of GM after topical steroid use. The fixed-effect model estimated the pooled recurrence rate, which was 11% (95% CI: 6–21%).

#### The recurrence rate following antibiotic use

**Fig. 3:**
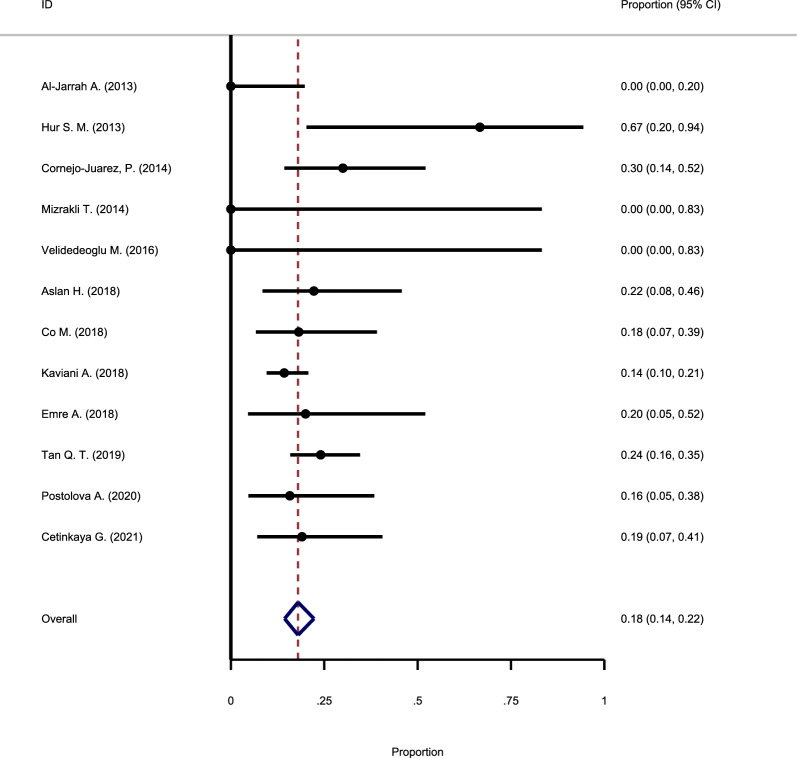
Forest plot showing the recurrence rate following antibiotic use

Twelve primary studies investigated the recurrence rate of GM after antibiotic use and were included in this meta-analysis. Analysis to assess the heterogeneity suggested that there is no evidence in favor of significant heterogeneity (Chi square = 0.3, I-square = 2.31%), and the fixed-effect model was used to pool the results of primary studies. The results suggested that the recurrence rate of GM after antibiotic use is 18% (95% CI: 14–22%) (Fig. 3).

#### The recurrence rate following MTX use

**Fig. 4:**
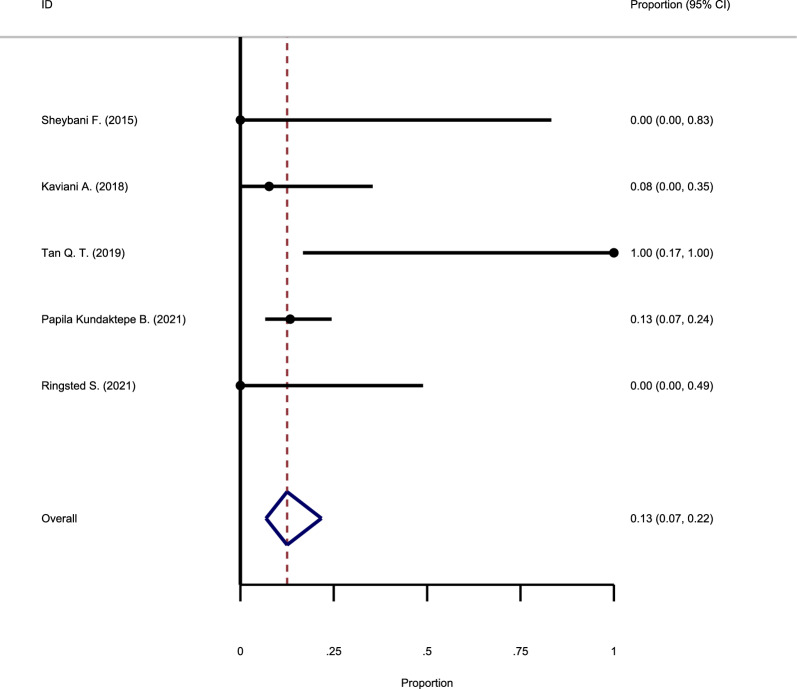
Forest plot showing the recurrence rate following Methotrexate use

Five studies investigated the recurrence rate after methotrexate use, and their results were pooled to estimate the overall recurrence rate. The analysis suggested no evidence in favor of significant heterogeneity between studies (Chi square = 0.0, I-square = 0%), and the fixed-effect model was used. The pooled recurrence rate was estimated as 13% (95% CI: 7–22%) (Fig. 4).

#### The recurrence rate following methotrexate and oral steroid use

**Fig. 5:**
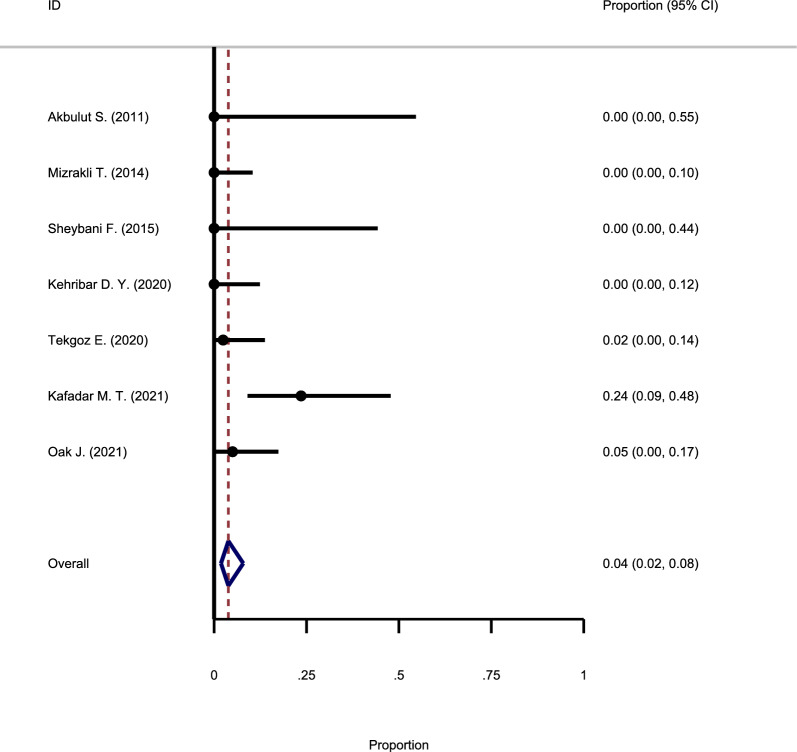
Forest plot showing the recurrence rate following methotrexate and oral steroid use

As shown in Fig. 5, seven studies met the inclusion criteria, which assessed the recurrence rate of GM following the combination of methotrexate and oral steroid use. The evidence revealed no substantial heterogeneity among studies, and the fixed-effect model was used to pool the reported primary results (Chi square = 3.97, I-square = 16.04%). The meta-analysis showed that the recurrence rate of GM after MTX and oral steroid use is 4% (95% CI: 2–8%).

#### The recurrence rate following antibiotic and steroid use

**Fig. 6:**
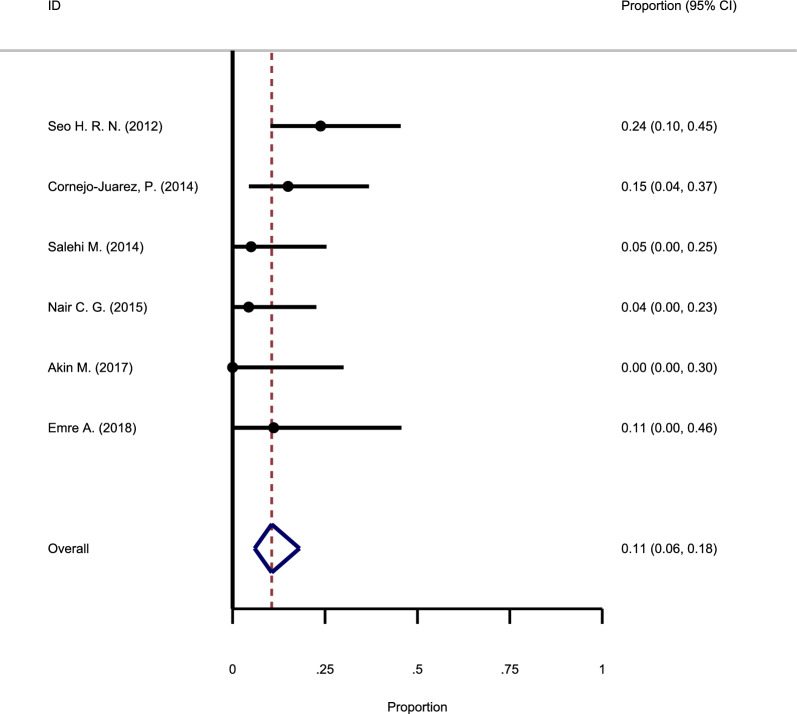
Forest plot showing the recurrence rate following antibiotic and steroid use

As shown in Fig. 6, six studies investigated the relapse rate after taking antibiotics and steroids. The heterogeneity between the studies showed no fundamental heterogeneity; therefore, the fixed-effect model was used to pool the primary findings (Chi square = 0.24, I-square = 18.6%). Based on the results of the meta-analysis, it was found that the summary recurrence rate of GM in this group is equal to 11% (95%CI: 6–18%).

#### The recurrence rate following observation

**Fig. 7:**
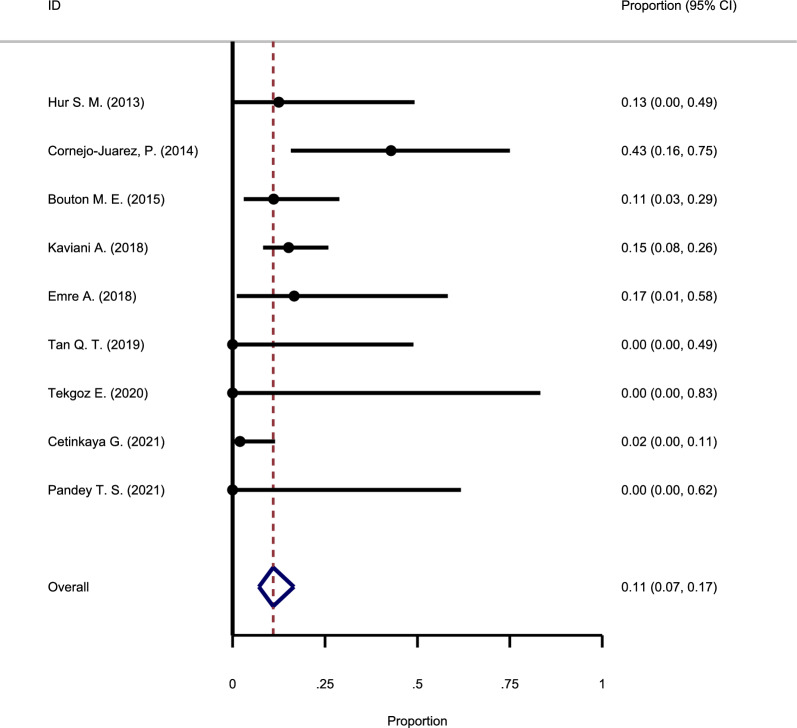
Forest plot showing the recurrence rate following observation

Among the retrieved articles, nine investigated the recurrence rate in the group that did not receive any intervention and were only under observation. As in the previous cases, the results of examining the heterogeneity among these nine studies showed no noteworthy heterogeneity between the studies (Chi square = 1.55, I-square = 12.66%). The overall recurrence rate in this group of articles was estimated at 11% (95% CI: 7–17%) based on the meta-analysis results of these studies (Fig. 7).

#### The recurrence rate following drainage

**Fig. 8:**
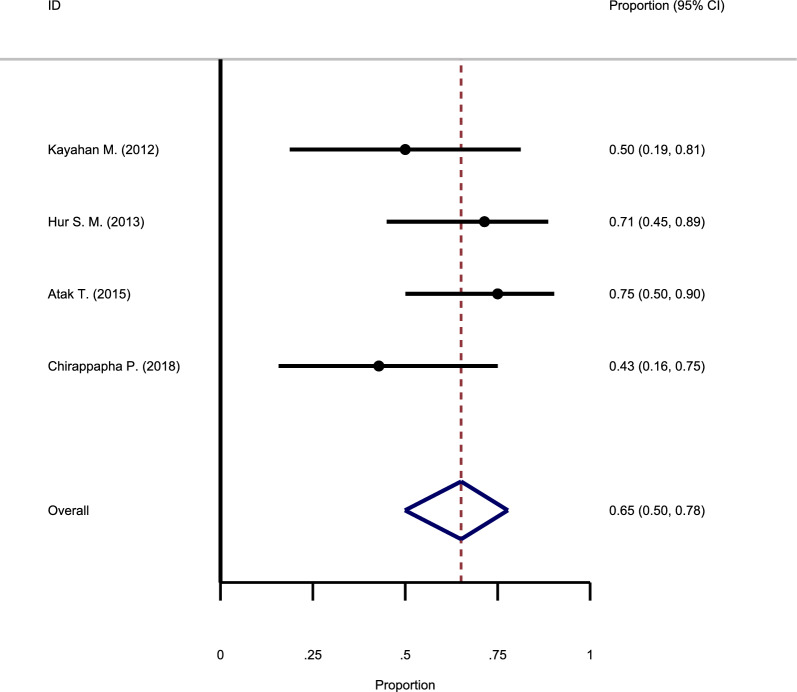
Forest plot showing the recurrence rate following drainage

In four studies, the drainage method was used to treat GM. Investigation of the articles’ heterogeneity showed no substantial heterogeneity between the studies (Chi square = 0.0, I-square = 0.0%). The meta-analysis results showed that the recurrence rate of GM after drainage is 65% (95% CI: 50–78%) (Fig. 8).

#### The recurrence rate following excision

**Fig. 9:**
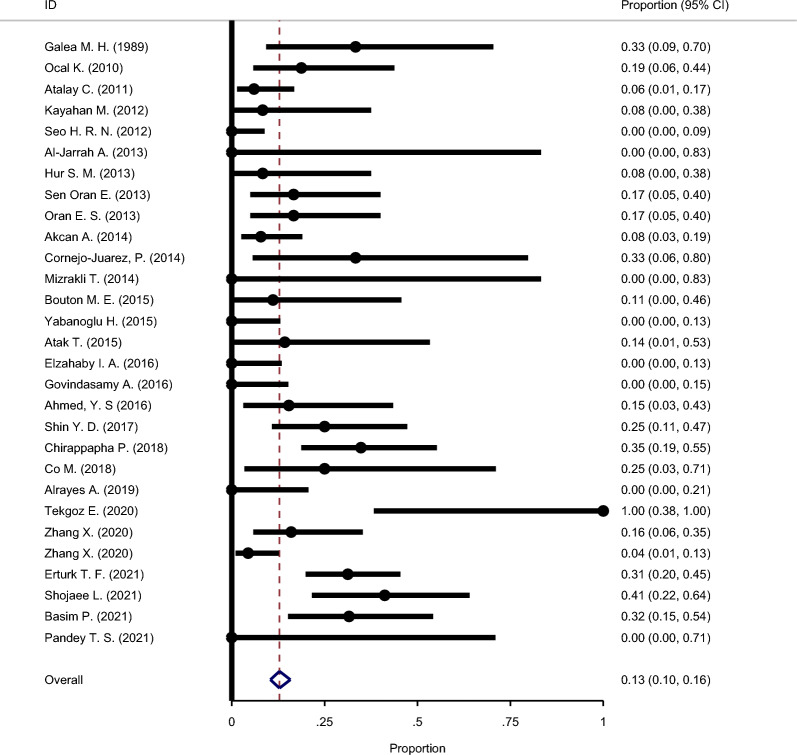
Forest plot showing the recurrence rate following excision

Figure 9 shows the results of the meta-analysis of GM recurrence rate after excision. Twenty-nine studies were included in this analysis. Based on the analysis, there was no considerable heterogeneity among the included studies (Chi square = 36.59, I-square = 32.07%). After integrating the results of the primary studies, the overall relapse rate in this treatment group was estimated at 13% (95% CI: 10–16%).

#### The recurrence rate following antibiotic use and surgery

**Fig. 10:**
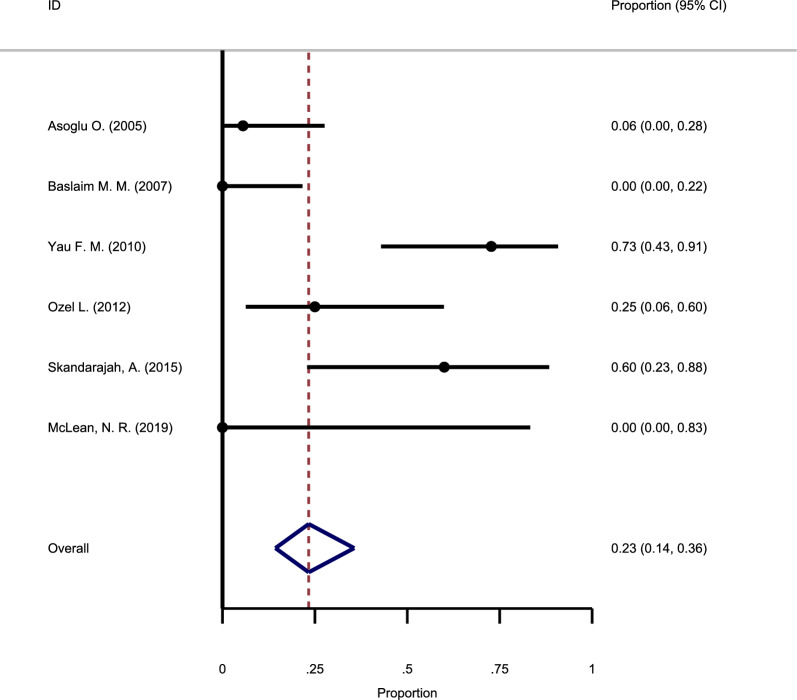
Forest plot showing the recurrence rate following antibiotic use and surgery

As shown in Fig. 10, six studies have investigated GM's recurrence rate after surgery and the use of antibiotics. No substantial heterogeneity was observed among these studies, and the fixed-effects model combined the findings (Chi square = 15.0, I-square = 30.96%). The meta-analysis results in this group showed a recurrence rate of 23% (95% CI: 14–36%).

#### The recurrence rate following steroid use and surgery

**Fig. 11:**
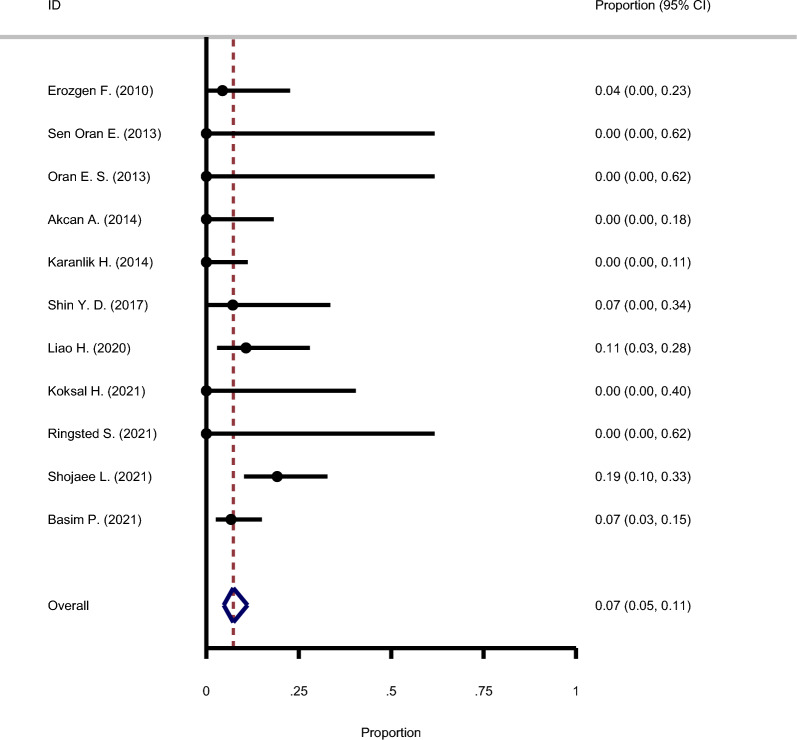
Forest plot showing the recurrence rate following steroid use and surgery

Eleven studies, as illustrated in Fig. 11, have looked into the recurrence rate of GM after surgery and steroid therapy. There was no significant heterogeneity across these studies, and the fixed-effects model was utilized to combine the results (Chi square = 3.43, I-square = 14.8%). According to the meta-analysis results, the overall recurrence rate in this group was 7% (95% CI: 5–11%). A summary of the recurrence rates for therapeutic modalities in GM is provided in Table 2.

**Table 2:** Recurrence rates for different therapeutic approaches in GM

Therapeutic method	Recurrence rate% (95% CI)	Number of studies
Oral Steroid	24 (21–27)	31
Topical Steroid	11 (6–21)	11
Antibiotic	18 (14–22)	12
MTX	13 (7–22)	5
MTX and Oral Steroid	4 (2–8)	7
Antibiotic and Steroid	11 (6–18)	6
Observation	11 (7–17)	9
Drainage	65 (50–78)	4
Excision	13 (10–16)	29
Antibiotic and Surgery	23 (14–36)	6
Steroid and Surgery	7 (5–11)	7

*MTX* methotrexate

### Risk of bias across the studies

Begg test was used to check the publication bias across studies. The findings proposed no evidence of publication bias in some meta-analyses, such as oral steroid use (Begg’s test *p*-value = 0.101). However, there was evidence of publication bias in others, such as excision (Begg’s test *p* value = 0.041).

## Discussion

### GM therapeutic methods

Therapeutic management of GM remains controversial, and there is limited evidence to guide treatment decisions. Healing of the disease with no recurrence in patient follow-up constitutes effective therapy of GM [76]. Surgical excision, including wide surgical resection, mastectomy with or without rapid breast reconstruction, frequent abscess drainage, antibiotics, topical or systemic corticosteroids treatment, immunosuppression with MTX, and close observation are among the treatment options [15, 37, 41, 43]. Several studies suggest that the disease will improve by itself through Observation alone [29, 30]. Some other studies consider conservative treatments the most effective treatment with the lowest recurrence rate [45, 55, 56, 70], while others find surgery the most appropriate treatment [1, 60, 62, 65]. Some studies believe that a combination of conservative and surgical therapy should be administered to decrease the recurrence rate [22, 42, 59, 77]. In our study, drainage had the highest recurrence rate, while the lowest rate was related to concurrent MTX and steroid treatment.

Observation and topical steroid treatment have demonstrated a lower recurrence rate than surgery and steroid therapy; this may be because observation and topical steroid therapy have been typically employed in mild occurrences of the condition. Since GM is a transient, benign illness that resolves by itself, observation may be an appropriate option in these instances, and other treatments should be used less frequently because of their side effects. Antibiotics are commonly administered due to the uncertainty of the diagnosis of GM prior to histopathological analysis; however, antibiotic therapy has been demonstrated to be ineffective for GM treatment since it is a sterile condition [78]. Treatment with systemic steroids is over six months, and adverse effects such as gaining weight, hyperglycemia, hypertension, Cushing syndrome, and an immunocompromised state may develop [31]. Furthermore, incorrect operation scheduling usually results in distorted breast appearance, diminishes the beauty of the breasts, and exacerbates patients' psychological burden and social difficulties [79]. Therefore, in noncomplicated cases, it is recommended to employ topical medications or observation, which consists of clinically and radiologically following patients at frequent intervals (1–3 months) (25).

In patients with diffuse breast inflammation, medical treatment is often preferred [11]. In our study, MTX had the lowest relapse rate among medical treatment options. However, patients often receive MTX as a second-line therapy, and it has rarely been administered as a monotherapy. MTX may be added to corticosteroids if symptoms do not improve, unfavorable steroid effects occur, or relapse is seen [73]. In the current study, MTX significantly lowered the recurrence rate when combined with corticosteroids. There are studies documenting MTX's efficacy in preventing disease relapse, lessening corticosteroid dose, and inducing disease remission [2, 7, 80]. Therefore, in extensive inflammation, the combination of MTX and corticosteroid can be used to lower the risk of Recurrence and the necessity for mastectomy, as well as eliminating the requirement for high doses of corticosteroid, thereby minimizing the adverse effects. In addition, consistent with the findings of Kafadar et al., because of the low rate of Recurrence in the therapy with corticosteroids and MTX, this combination can be employed after Recurrence with other treatments, including surgery [41]. However, due to the higher prevalence of GM while breastfeeding and the adverse effects of MTX on the infant during breastfeeding [81, 82], it should be noted that MTX should be avoided in breastfeeding mothers in favor of corticosteroid monotherapy.

Despite the stated high recurrence rate of drainage, this treatment is unavoidable when an abscess is formed [49, 56]. Antibiotics can be beneficial in these situations and significantly decrease the recurrence rate. Similar findings from previous studies indicate that antibiotics can be given empirically for 7–10 days if an abscess occurs [83].

In recent years, nonsurgical treatment options, such as medicines, have surpassed surgical treatment. A limited surgery technique is associated with a high risk of relapse, whereas negative margin surgery is associated with significant side effects, such as cosmetic concerns. However, excision is advised when the differential diagnosis between GM and cancer is uncertain [53]. Additionally, surgical excision may be beneficial if the inflammation is localized [11, 31]. The combination of surgery and corticosteroids is an effective treatment for GM, and due to the low recurrence rate, it could be utilized as the first line in these situations. Moreover, steroids given to patients with extensive lesions before surgery may reduce the lesion’s size, improving the procedure’s cosmetic outcome [65]. However, it is essential to note that since corticosteroids slow down wound healing, the minimum effective dose should be used until the wound heals after surgery.

### Rifampicin therapy in GLM

In the past decade, there has been a noticeable change in the literature about the management of GLM, shifting from surgery to medicinal treatment. Many studies have identified *Corynebacterium kroppenstedtii* as the principal pathogenic component of GLM, although other atypical pathogens such as *Pseudomonas oleovorans*, *Acinetobacter baumannii*, and *Thermus thermophilus* may also be intimately associated with GLM. Abnormal levels of prolactin hormone and autoimmune dysfunction are significant causes of GLM. Each of the three pathogenic variables can act alone as the cause of GLM or combine to promote the development of GLM. Therefore, lipophilic antibiotics such as Rifampicin, and prolactin inhibitors could be effective treatments [84].

A study by Farouk et al. demonstrated the effectiveness of a Rifampicin therapy regimen of 300 mg twice daily for 6–9 months in treating GLM. The treatment resulted in complete clinical and ultrasonographic response in 30 patients, with no recurrent episodes during a median follow-up of 15.5 months (average 3–35 months). This suggests that Rifampicin could be an effective standalone medical treatment option for GLM, replacing the need for surgery or corticosteroids [15].

In a recent clinical trial, Zhou et al. demonstrated the safety and efficacy of Rifampicin-based triple therapy (Rifampicin, Isoniazid, and Ethambutol) in treating 82 patients with GLM. The treatment continued for a median of 8 months, and 8 patients (9.76%) experienced a relapse [85].

Therefore, special attention should be paid on utilizing this drug for treating GLM. Moreover, trials should prioritize using rifampicin for more precise outcomes.

### Limitations

One of the limitations of this study is that since most studies were retrospective or used non-randomized sampling, there is a risk of bias in comparing the recurrence rate of each treatment. Moreover, drug use dosage and duration vary between studies, and a particular drug dose has not been established. Another limitation of the study is the dispersion in the duration of follow-up across different studies. The duration of follow-up depends on factors such as the severity of the condition, the effectiveness of the treatment, the presence of any complications, and the individual’s overall progress and healing [12, 30]. There is no specific duration for patient follow-up. However, given the substantial variations in severity of the disease and follow-up durations, there is a potential for inaccuracies in comparing the recurrence rate associated with each therapeutic approach. In addition, a number of the treatments in the studies had small sample sizes, which might lead to estimation errors when comparing the effectiveness of the treatment to other treatment methods.

## Conclusion

GM is a rare, benign breast disease without a specific treatment strategy. Clinicians should choose the treatment modality based on the patient’s characteristics and disease complications. The results of this study show that combination therapy is superior to monotherapy in reducing the risk of Recurrence. Some patients with mild symptoms may only require observation or topical treatment. Abscesses could well be treated with drainage in conjunction with antibiotics. Surgical excision combined with steroid therapy is an option for patients with localized lesions like masses. Oral steroids, combined with MTX, could be the first line of treatment for patients exhibiting more severe symptoms, such as widespread breast swelling or acute skin inflammation. It should be noted that the adverse effects of the treatments must be explained to the patients, and the patient's preferences must be incorporated into the treatment. In summary, it can be stated that the treatment of GM varies depending on the situation, and it is required to develop guidelines based on the present study and other similar studies so that the appropriate treatments can be administered focusing on the disease’s features.

## Data Availability

All data for the analyses are presented in Table 1.

## Abbreviations

PRISMA: Preferred reporting items for systematic reviews and meta-analyses
GM: Granulomatous mastitis
CI: Confidence interval
MeSH: Medical subject headings
IGM: Idiopathic GM
SGM: Specific GM
CGM: Chronic granulomatous mastitis
GLM: Granulomatous lobular mastitis
TM: Tuberculous mastitis
MTX: Methotrexate
